# Adaptive forgetting speed in working memory

**DOI:** 10.3758/s13423-024-02507-2

**Published:** 2024-05-08

**Authors:** Joost de Jong, Sophia Wilhelm, Elkan G. Akyürek

**Affiliations:** Experimental Psychology, University of Groningen, Grote Kruisstraat 2/1, Groningen, the Netherlands

**Keywords:** Working memory, Forgetting, Probing hazard

## Abstract

Working memory is known to be capacity-limited and is therefore selective not only for what it encodes but also what it forgets. Explicit forgetting cues can be used effectively to free up capacity, but it is not clear how working memory adaptively forgets in the absence of explicit cues. An important implicit cue that may tune forgetting in working memory is the passage of time. When information becomes irrelevant more quickly, working memory should also forget information more quickly. In three delayed-estimation experiments, we systematically manipulated how probing probability changed as time passed on after encoding an item (i.e., the “probing hazard”). In some blocks, probing hazard decreased after encoding an item, requiring participants to only briefly retain the memory item. In other blocks, the probing hazard increased or stayed flat, as the retention interval was lengthened. In line with our hypothesis, we found that participants adapted their forgetting rate to the probing dynamics of the working memory task. When the memory item quickly became irrelevant (“decreasing” probing hazard), forgetting rate was higher than in blocks where probing hazard increased or stayed flat. The time course of these adaptations in forgetting implies a fast and flexible mechanism. Interestingly, participants could not explicitly report the order of conditions, suggesting forgetting is implicitly sped up. These findings suggest that implicit adaptations to the temporal structure of our environment tune forgetting speed in working memory, possibly contributing to the flexible allocation of limited working memory resources.

## Introduction

Working memory has clear capacity limits (Cowan, 2001). As a consequence, working memory is not only selective about what it encodes, but also selective about what it forgets (Souza & Oberauer, 2016). Humans and non-human animals can use explicit cues to drop irrelevant information in order to “free” capacity (Dames & Oberauer, 2022; Williams et al., 2013; Williams & Woodman, 2012). Information that has been cued to forget is remembered less well, or even completely lost, and subsequent information is encoded better as a result of this cleared capacity (Dames & Oberauer, 2022; Williams et al., 2013), provided that forgetting cues are highly reliable (Williams & Woodman, 2012).

Reliable, explicit cues to forget information, however, might be rare in real-life scenarios. Yet, there are numerous implicit cues that inform us about the relevance of information in our environment. For instance, an important implicit cue to forget is the passage of time. As time passes on, information typically becomes less relevant to our current goals. Crucially, in some situations, information becomes irrelevant more quickly than in others. Consider reading an article. When trying to understand the full scope of an article, individual pieces of information stay relevant throughout our read. Conversely, browsing that same article for a specific quote does not require us to maintain each sentence we checked. Hence, an important question is whether forgetting mechanisms in working memory can adapt to these implicit temporal regularities, as has also been found when encoding information (de Jong et al., 2023). That is, can humans detect implicit cues that information becomes irrelevant more quickly, and speed up their forgetting accordingly?

There is some evidence to suggest that humans can adapt their rate of forgetting to how quickly information becomes irrelevant. Anderson and Schooler (1991) demonstrated that, for a variety of real-life scenarios, the probability that a piece of information re-occurs in the future declines over time. The authors hypothesize that forgetting in memory systems adapts to these temporal features of the environment. In a series of experiments, Anderson et al. (1997) empirically confirmed that working memory adapts to the rate at which information becomes irrelevant. When the probability that a verbal item would be probed declined over time (i.e., when the hazard rate of the probe declined), participants forgot that item more quickly. However, it is not clear whether these results reflect adaptations in working memory per se or a gradual reallocation of attention to the secondary articulatory suppression task that Anderson and colleagues (Anderson et al., 1997) employed simultaneously. It is also not clear whether their results also apply to visual working memory.

**Fig. 1:**
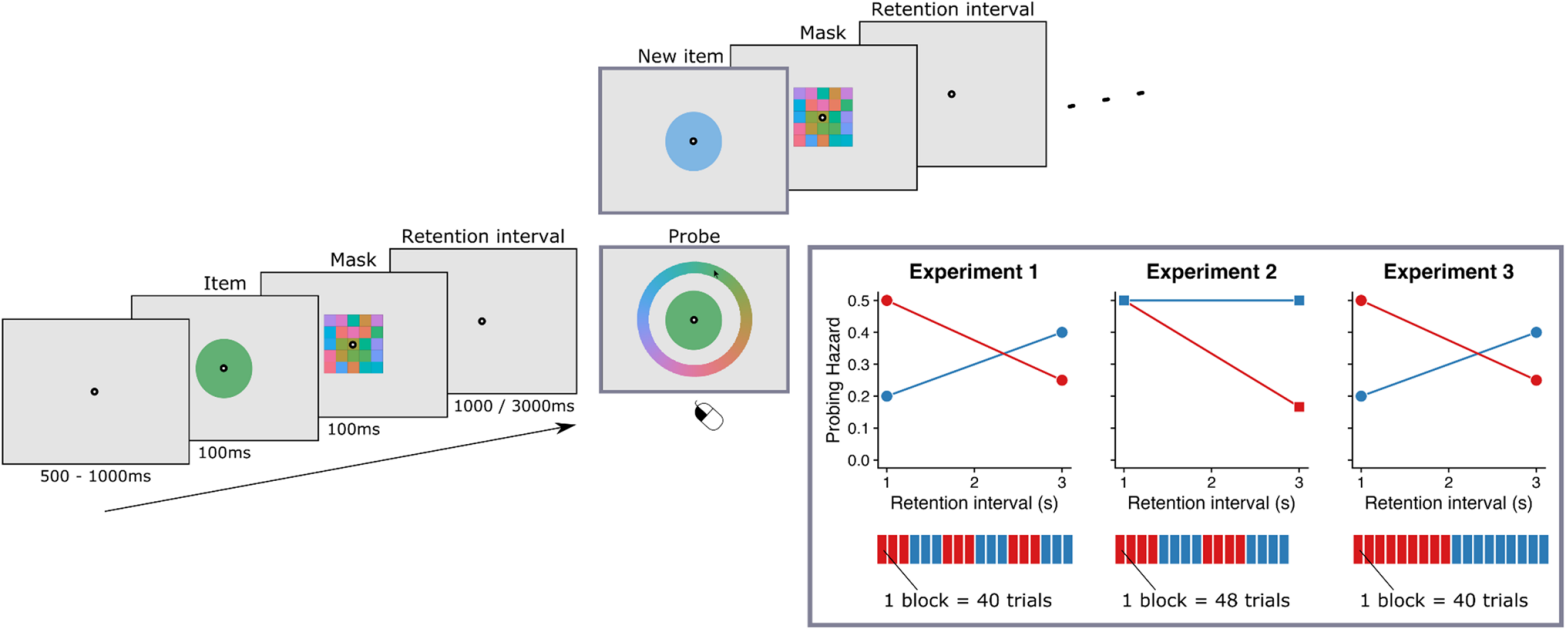
Overview of the experimental set-up. Participants were presented with a single memory item (colored circle), which was followed by a mask to disrupt the afterimage. After a retention interval (1,000 or 3,000 ms) participants were either (1) probed by a color wheel, where they reproduced the memory item, or (2) presented with a new memory item, after which the trial proceeded. Importantly, the hazard that an item would be probed was either increasing, constant or decreasing as time passed on after encoding an item, and this was varied between consecutive sets of blocks (mini-blocks)

In three delayed-estimation experiments without a concurrent task, we tested whether forgetting in visual working memory adapts to how quickly information becomes irrelevant. In our experiment, we manipulated the time course of the probing hazard by sometimes not probing the item currently in memory but presenting a new item that had to be remembered instead (Fig. 1). In some blocks, probing hazard increased or stayed constant, while in others it decreased as time passed on after encoding an item. That is, in “decreasing” blocks, it was very likely that a color wheel probe would appear one second after encoding an item, but as time passed on, this probability dropped, making it more likely that a *new* item needed to be encoded (and vice versa in “increasing” blocks). We hypothesized that if forgetting in working memory is adaptive, then the steeper the drop in probing hazard, the faster the forgetting. To preview our results, we found that participants implicitly sped up forgetting when information became irrelevant more quickly.

## Methods

### Participants

Participants were first-year psychology students (mean age = 20.1 years; 73% female) at the University of Groningen who participated in exchange for course credit. On the basis of a checklist developed by the EC-BSS at the University of Groningen, the study was exempt from full ethical review. The study was conducted in accordance with the ethical standards of the 1964 Helsinki Declaration. Some participants did not perform the task well. Therefore, participants whose mean absolute error was higher than Q3 (third quartile) + 3 x IQR were excluded from further analysis (criterion was determined per experiment). No participants were excluded in Experiment 1, one in Experiment 2, and two in Experiment 3.

### Apparatus and stimuli

The experiment was programmed using OpenSesame, a freely available software tool for designing experiments (Mathôt et al., 2012). Stimuli were presented on a 19-in. CRT screen with a resolution of 1,280 x 1,024 pixels, refreshing at 100 Hz. The testing room was sound-attenuated with dimmed lights and participants were seated approximately 60 cm away from the screen. A gray background was maintained throughout the experiment. Memory items were colors presented centrally on the screen. Colors were presented in CIElab color space (L* = 70, a = 0, b = 0, radius = 38). Memory items were followed by a mask to disrupt the retinal or cortical afterimage. The mask consisted of a square containing 25 individual squares in a 5 x 5 grid each filled with a different color randomly sampled from the CIElab color space.

### Procedure

Participants signed written informed consent and were given verbal instructions. They then performed several practice trials before moving on to the experimental trials. On any given trial, participants were presented with a fixation dot for 500–1,000 ms (uniformly sampled), after which the memory item was presented for 100 ms. Immediately following this, a mask was presented for 100 ms to eliminate any retinal or cortical after-image. The reason for using such a brief presentation time was to create conditions where *preemptive* forgetting was beneficial. If participants get ample time to encode the new item, there would be no need to forget the previous item (e.g., at longer encoding times, participants might have time to *actively* disregard information before encoding the next item). Once the mask was removed, the delay period started, during which the participants had to keep the memory item in mind. The duration of the retention interval was either 1,000 or 3,000 ms. After the retention interval, one of two things happened: (1) a randomly rotated color wheel was presented, prompting participants to report the color with a mouse click, or (2) a new color would be presented that they needed to store in memory and the trial proceeded (i.e., after 1,000 or 3,000 ms the new color would either be probed, or yet another item would be presented, etc.). This allowed us to systematically vary how probing hazard evolved after item presentation. In some blocks, probing hazard decreased (“decreasing” blocks), and in some it increased (“increasing” blocks), or alternatively, stayed flat (“flat” blocks).

Once participants indicated their response, feedback was provided by assigning points as a function of absolute error. The score was 0 when |error| > 45°. Between 45 and 0°, the score scaled linearly with absolute error from 0 to 100 points. At the end of each block, participants were presented with their cumulative score of points acquired during the block, and their block-wise high score so far. Participants were able to set new high scores, as a means to motivate them to keep up their performance throughout the task. Block order was counterbalanced across participants, such that half of the participants started with a mini-block with “decreasing” probing hazard, and the other half with a mini-block with “increasing” (Experiments 1 and 3) or “flat” (Experiment 2) probing hazard (see Fig. 1).

## Experiment 1

In this initial experiment (N = 50), we tested whether forgetting was faster in blocks where probing hazard decreased, as opposed to blocks where probing hazard increased. For our first experiment, we did not know the effect size a priori, but we would have a power of 93% assuming a medium effect size (Cohen’s *d* = 0.5) for a paired-sample t-test. The time course of probing hazard within trials varied between blocks. In “decreasing” blocks, the overall probing probability was 60%, but the probing hazard decreased over time. That is, after 1 s of retention time, the probability that the item would be probed was .5, but after 3 s, the probability that the item would be probed was .25 (see Fig. 1). In “increasing” blocks, the probing hazard increased from 0.2 to 0.4. Note, however, that both retention intervals occurred equally often. Participants would switch between conditions every three blocks, each containing 40 trials. The order with which condition participants started was counterbalanced. In total participants completed 18 blocks, totaling 720 trials. Participants were not made aware of the manipulation of probing hazard.

## Experiment 2

The second experiment (N = 45) was performed to replicate the findings of the first experiment, while equating probing hazard after one second of retention time. Also, we wanted to enhance the effect by inducing a sharper drop in probing hazard for the “decreasing” blocks. Assuming at least the same effect size as in Experiment 1 (*d* = 0.35), the power was 76%. In blocks with “decreasing” probing hazard (overall probing probability = 58%), the probing hazard after one second was .5, which decreased to .167 at 3 s. In blocks with “flat” probing hazard, the overall probing probability was now 75%, but stayed flat at .5. As in Experiment 1, both retention intervals occurred equally often. Furthermore, we also slightly increased the duration of each block, so that each block now consisted of 48 trials. Also, participants completed four blocks before switching block type. The number of blocks stayed the same, but in total participants now completed 864 trials. As in the previous experiment, participants were not made aware of the manipulation of probing hazard.

## Experiment 3

The third experiment (N = 47) was aimed at checking whether forgetting rate adapted throughout an experiment, and whether those adaptations were implicit or explicit. Experiment 3 was the same as Experiment 1, except for two things. First, the order of “increasing” and “decreasing” blocks was such that participants started with nine consecutive blocks of the same condition (increasing/decreasing), and then switched to the other condition. Second, at the end of the experiment, we asked three questions to probe explicit knowledge of the switch between conditions. The first question was intended to elicit explicit knowledge spontaneously: “Did you notice that something had changed halfway through the experiment? If yes, please state (in either Dutch or English) what you think changed. If not, you can leave this question empty.” The second question gave away what had changed and asked participants whether they had indeed noticed this change: “In one half of the experiment, you needed to remember the color for a brief period of time before it was probed. In the other half of the experiment, you needed to remember the color for a longer time before it was probed. Did you notice this (Yes / No)?” The third question was aimed at checking whether participants had accurate knowledge about the order of conditions: “Can you indicate which order you experienced in the experiment? If you don’t know, please make a guess (First half: remember for a SHORT time. Second half: remember for a LONG time / First half: remember for a LONG time. Second half: remember for a SHORT time).”

### Analysis

All analyses were performed in R (R Core Team, 2018; version 4.2.2). The data of the three experiments were analyzed using the same approach. Trials where participants took longer than 10 s to respond were excluded from any analyses (0.1 % of all trials).

Absolute error angle was calculated as the absolute difference between the reproduced angle and the actual angle of the memory item (thus, *|error|* spans from 0 to 180°). Forgetting rate (in degrees per second) was calculated for each participant and each block type (i.e., “decreasing”, “flat”, “increasing”), by fitting a linear regression that predicted mean absolute error with retention interval as a predictor. The resulting coefficient for retention interval represents the forgetting rate in degrees per second, which was then used in subsequent analyses. The rationale for this approach was twofold. Firstly, we want to have an approximately normally distributed dependent variable to perform inference on. Absolute error angle is heavily skewed but forgetting rate less so. Secondly, we wanted to perform a “paired” analysis that takes into account that trials are grouped within participants. By calculating forgetting rate per participant, per condition, we take that grouping into account.

To assess whether forgetting rates differed between block types, we computed forgetting rates pooled across experimental blocks and performed a one-sided paired t-test. Comparisons between Experiments 1 and 2 were done with a linear regression, predicting forgetting rate with “hazard drop” (i.e., how quickly the hazard increased or decreased after encoding). For the linear regression we report t-values and p-values associated with the absolute t-value. In order to study how adaptation evolved over the course of the experiment, we computed the forgetting rate for each individual experimental block and performed between and within-subject comparisons using one-sided t-tests. In Experiment 3, we wanted to consider the entire time course of adaptation in the first and second half of the experiment. To this end, we fitted a linear regression model for even and odd participants, with experimental block as a predictor and forgetting rate as the dependent variable. The resulting coefficient represented how quickly forgetting rate increased. We also added the interaction with block type to assess whether the change in forgetting rate was different when switching to an increasing versus decreasing probing hazard. We report t-values and p-values associated with the absolute t-value. We also computed Bayes factors (BF_10_) for t-tests using the BayesFactor package (Morey & Rouder, 2022; version 0.9 12-4.4). We used the default Cauchy prior (*r* = 1/√2). One-sided tests were performed as described in Wetzels et al. (2009) by limiting the prior mass to the region (0, ∞). The reported BF_10_ reflects the ratio between the posterior probability for the alternative hypothesis that the standardized effect is bigger than zero (i.e., forgetting rate is larger in “decreasing” than “increasing/flat” blocks) and the null hypothesis that the standardized effect size is exactly zero. For linear regressions, we also compute BF_10_, where we compare the null model (i.e., “intercepts-only” model or “main effects only” models) with models including the main effect or interaction effect. The reported BF_10_ reflects the ratio between the posterior probability for the alternative and the null model.

## Results

### Information that outdates quickly is more quickly forgotten

**Fig. 2:**
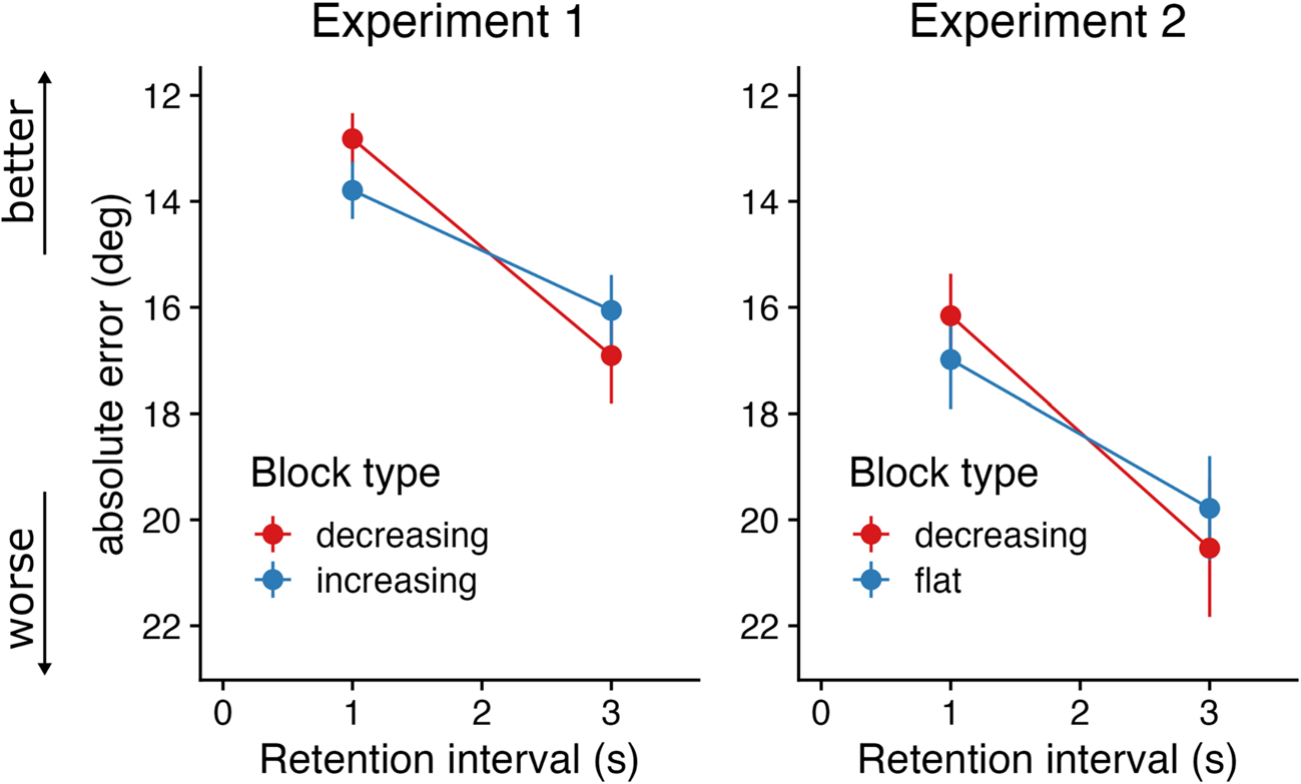
Results of Experiments 1 and 2. Mean |error| is plotted over retention interval for “decreasing” (red) and “increasing/flat” (blue) blocks. Note that all measures on the y-axis reflect performance, where higher is better (the axis for mean absolute error is flipped)

In Experiment 1, we found that forgetting was faster in “decreasing” blocks (*M* = 2.05 °/s, *SD* = 2.42), where information became outdated quickly, than “increasing” blocks (*M* = 1.13 °/s, *SD* = 1.38; Fig. 2; *t*(49) = 2.40, *p* = 0.010, BF_10_ = 4.06). Consistent with our hypothesis, this suggests that when information quickly became irrelevant (decreasing hazard), participants forgot that information more quickly compared to when information stayed relevant for a longer time (increasing hazard). In Experiment 2, forgetting rate in “decreasing” blocks (*M* = 2.19 °/s, *SD* = 3.25) was not significantly different from forgetting rate in “flat” blocks (*M* = 1.61, *SD* = 2.27; Fig. 2B; *t*(44) = 1.43, *p* = 0.079, BF_10_ = 0.77), although no strong evidence for the null hypothesis was obtained.

### Adaptive forgetting is driven by the rate at which information gets outdated

Why were the effects in Experiment 2 less pronounced than in Experiment 1? There were some small procedural differences between experiments (e.g., number of trials in an experimental block, number of trials per block type), but the main difference was how probing hazard evolved over a trial. In Experiment 1, probing hazard increased in some blocks, while in Experiment 2 probing hazard remained constant for some blocks (Fig. 1). Also, for “decreasing” blocks, the probing hazard dropped more steeply in Experiment 2. If forgetting rate adapts to the rate at which information becomes outdated, the slope of the hazard function should be the main driver of adaptive forgetting. More specifically, forgetting rate in the “flat” blocks should lie somewhere between the “decreasing” and “increasing” blocks, while the “decreasing” blocks of Experiment 2 should be at least as high as the “decreasing” blocks of Experiment 1. To this end, we computed the “hazard drop” (i.e., how quickly the hazard increased or decreased per second) for each condition in each experiment and tested its effect on forgetting rate

**Fig. 3:**
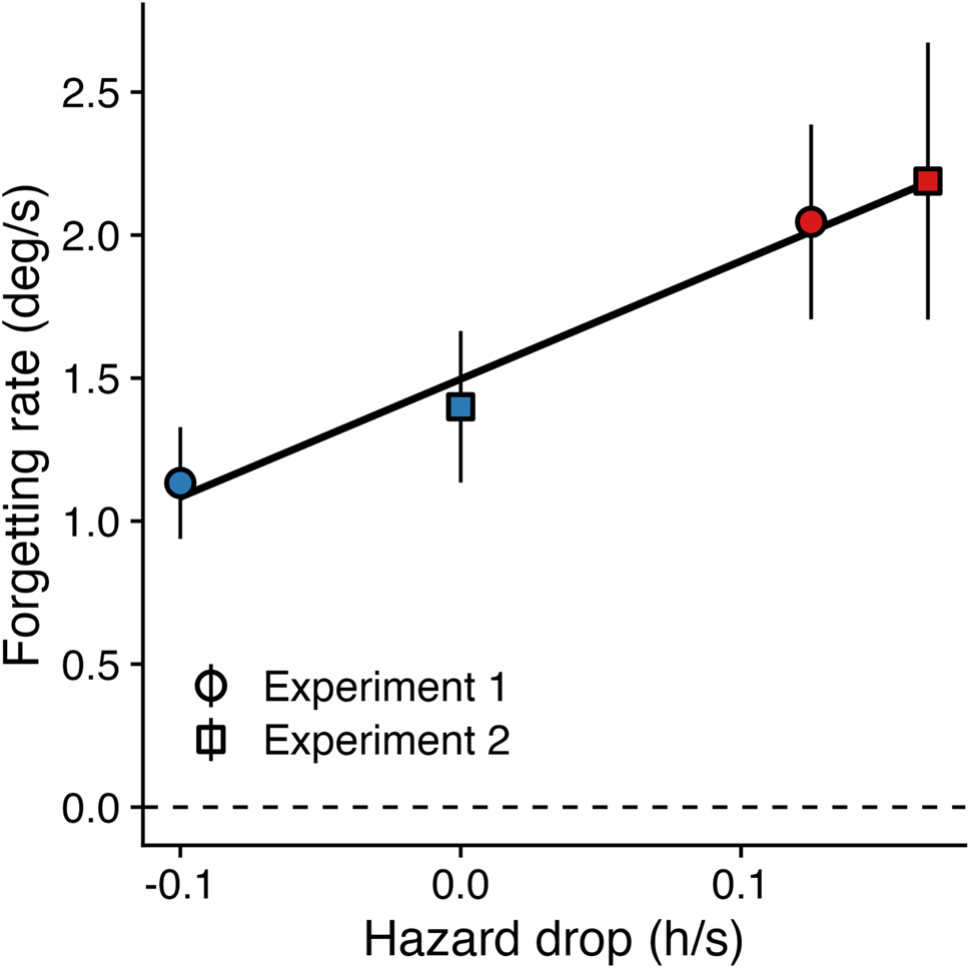
Analysis across experiments. For Experiments 1 (circles) and 2 (squares), the forgetting rate per block type is plotted for each hazard drop (in hazard rate per second)

We found that forgetting rate increased with hazard drop (Fig. 3; β = 4.13, *SE* = 1.57, *t* = 2.64, *p* = 0.009, BF_10_ = 3.87). This suggests that discrepancies between Experiment 1 and 2 can be partly explained by differences in how probing hazard evolves over time. In sum, this again demonstrates a link between the temporal dynamics of the working memory task and the temporal dynamics of forgetting.

### Adaptations in forgetting rate are fast and flexible

How is adaptive forgetting learned over the course of the experiment? In order to study the time course of adaptations in forgetting, we computed forgetting rates (based on |error|) for each experimental block, for each participant. This allows us to do a comparison of the forgetting rate between participants who started with “decreasing” blocks and participants who started with “increasing” or “flat” blocks. In Experiment 1, forgetting was significantly higher in the “decreasing” blocks compared to the “increasing” blocks at the third experimental block of the experiment (*t =* 3.41, *df =* 39.494, *p* < 0.001, BF_10_ = 49.80), just before switching block type. These results suggest that participants learned to forget relatively quickly (also see, Anderson et al., 1997). In Experiment 2, participants in the “decreasing” short block did not show more forgetting statistically than participants in the “flat” block in the fourth experimental block (*t =* 1.39, *df* = 34.409, *p* = 0.09, BF_10_ = 1.00), just before switching block type.

**Fig. 4:**
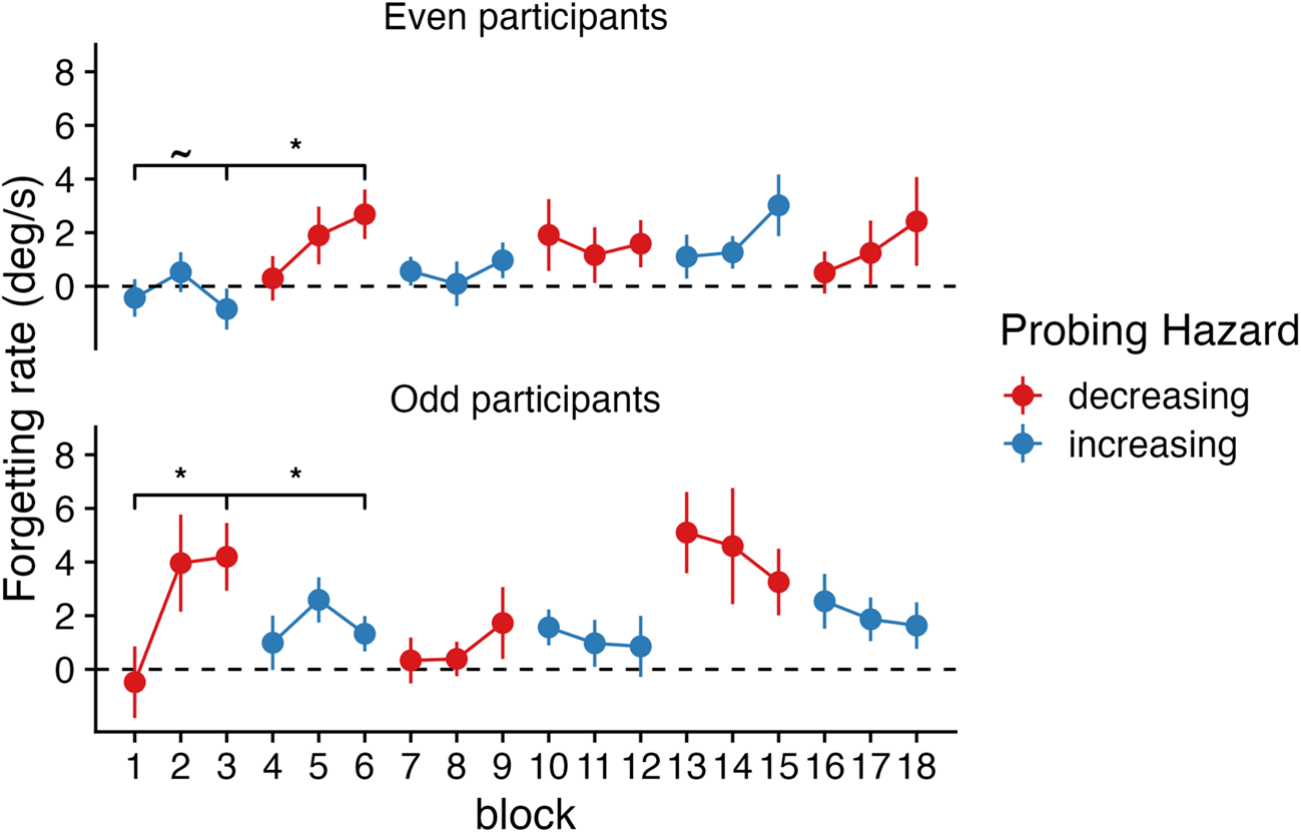
Time course of forgetting rates in Experiment 1, divided into even and odd participants, who started with different block types. Brackets represent the significance of paired-samples t-tests, representing (absence of) adaptation between block 1 and block 3, and re-adaptation between block 3 and block 6

We also tested for adaptation within participants. Using paired t-tests, we checked (1) whether achieved levels of adaptation could be unlearned, and (2) whether forgetting rate could still be adapted after having experienced several blocks with “increasing” or “flat” hazard rates. In Experiment 1, we found that participants who started with three “decreasing” blocks (odd participants) showed significant adaptation (block 1 vs 3; *t*(24) = 2.73, *p* = 0.005, BF_10_ = 8.36; see Fig. 4). Moreover, those same participants significantly unlearned their achieved levels of adaptation over the course of three “increasing” blocks (block 3 vs. block 6; *t*(24) = -2.19, *p* = 0.019, BF_10_ = 3.10). Conversely, participants who started with three “increasing” blocks (even participants), did not show statistically significant adaptation after three “increasing” blocks (block 1 vs. 3; *t*(24) = -0.41, *p* = 0.687, BF_10_ = 0.23). However, they were still able to adapt their forgetting rate after three “decreasing” blocks (block 3 vs. 6; *t*(24) = 2.75, *p* = 0.006, BF_10_ = 8.67). These findings demonstrate that adaptation of forgetting rate in working memory can be relatively fast and flexible.

**Fig. 5:**
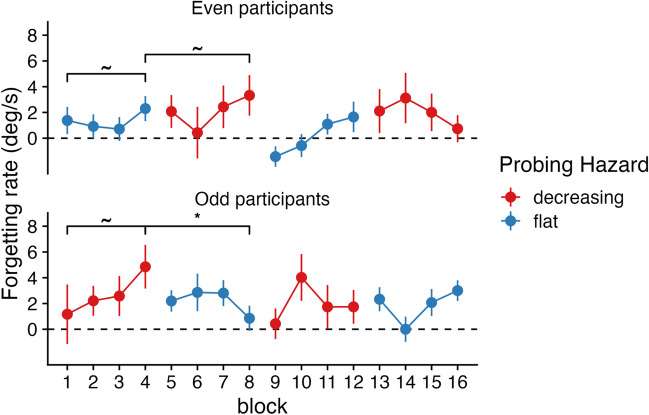
Time course of forgetting rates in Experiment 2, divided into even and odd participants. Brackets represent the significance of paired-samples t-tests, representing (absence of) adaptation between block 1 and block 4, and re-adaptation between block 4 and block 8

The results of Experiment 2 were less convincing. We found that participants who started with four “decreasing” blocks (odd participants) did not show significant adaptation (block 1 vs. 4; *t*(22) = 1.13, *p* = 0.13, BF_10_ = 0.66; see Fig. 5). However, those same participants significantly unlearned their achieved levels of adaptation over the course of four “flat” blocks (block 4 vs. block 8; *t*(22) = -2.12, *p* = 0.023, BF_10_ = 2.75). Participants who started with four “flat” blocks (even participants), did not show statistically significant adaptation after four “flat” blocks (block 1 vs. 4; *t*(21) = 0.73, *p* = 0.47, BF_10_ = 0.28), but they also did not increase their forgetting rate after four “decreasing” blocks (block 4 vs. 8; *t*(21) = 0.55, *p* = 0.29, BF_10_ = 0.36).

### Forgetting rate adapts throughout the experiment

**Fig. 6:**
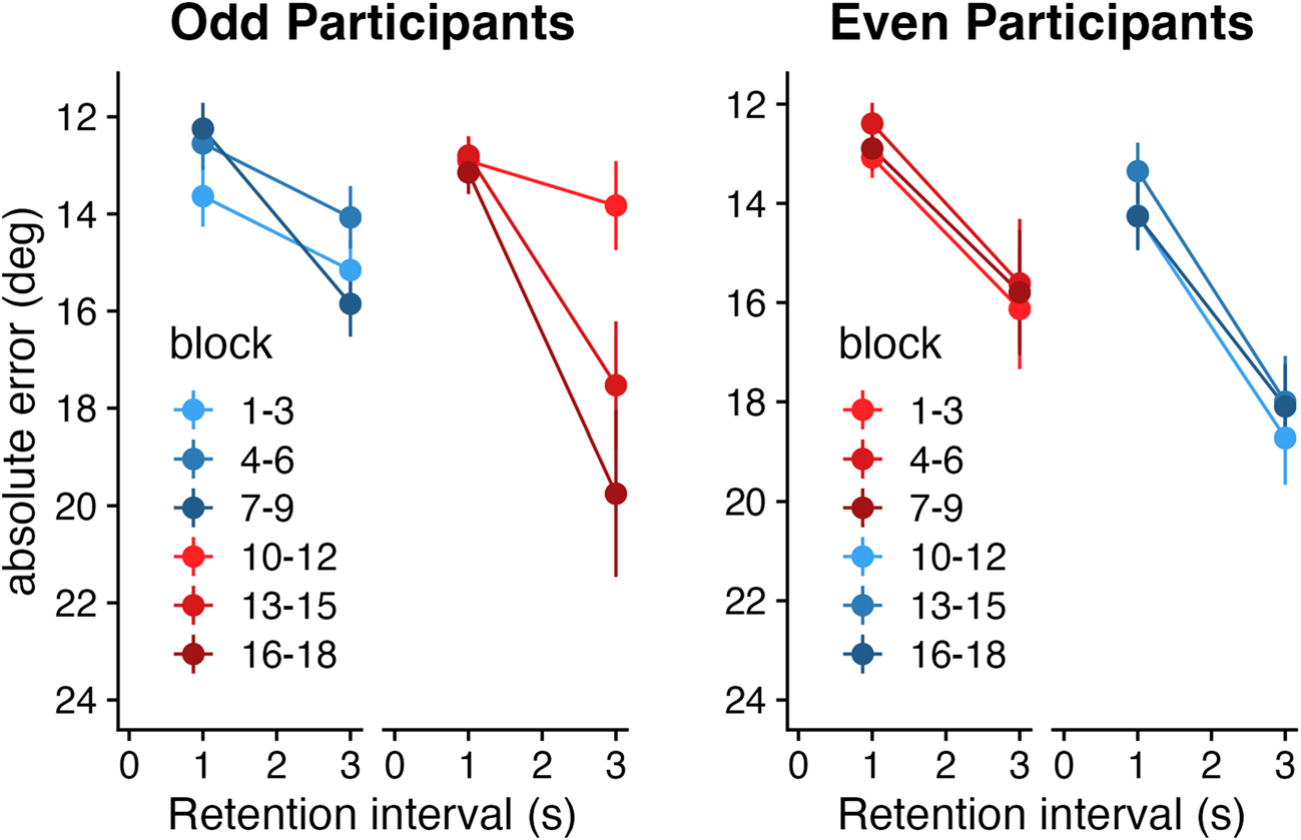
Forgetting over the course of Experiment 3. Absolute error is plotted as a function of retention interval. **Left panel:** Odd participants start out with nine consecutive blocks with an increasing probing hazard (shades of blue; left). Then, they switch to a decreasing probing hazard (shades of red; right). **Right panel:** Even participants start out with nine consecutive blocks with a decreasing probing hazard (shades of red; left). Then, they switch to an increasing probing hazard (shades of blue; right). Note that all measures on the y-axis reflect performance, where higher is better (the axis for mean absolute error is flipped)

As we have seen in Experiments 1 and 2, adaptations in forgetting rate are relatively fast and flexible. However, as the experiment progressed, differences in conditions became less and less apparent, possibly due to lingering effects of previous blocks. In Experiment 3, we set out to study whether forgetting rate adapts throughout the experiment (see Fig. 6). Participants were exposed to nine consecutive blocks of decreasing or increasing probing hazard, and then switched halfway through the experiment. Contrary to our expectations, forgetting rate did not differ between block types in the first half of the experiment (*t(*354.85) = 0.92, *p* = 0.35, BF_10_ = 0.27). However, after switching to a decreasing probing hazard, participants steadily increased their forgetting rate (β = 0.47 (forgetting rate per block), *t* = 3.01, *p* = 0.003, BF_10_ = 9.90), while participants switching to an increasing probing hazard maintained a stable forgetting rate (β = -0.04 (forgetting rate per block), *t* = -0.43, *p* = 0.738, BF_10_ = 0.16). This change in forgetting rate was statistically different between conditions (*t* = 2.55, *p* = 0.011, BF_10_ = 3.48). Notably, while forgetting rate increased for odd participants, performance at the 1-s retention interval remained stable. This suggests that not encoding, but specifically forgetting was adapted to the new task demands. Odd participants started with nominally higher forgetting rates than even participants. However, unlike Experiments 1 and 2, they were unable to slow down their forgetting after switching to an increasing probing hazard. These findings suggest that it may be easier to “learn to forget” than to “forget to forget”.

### Adaptations in forgetting rate are largely implicit

We did not inform participants about manipulations of probing hazard in any of the experiments, suggesting that participants did not need explicit instructions to adapt their forgetting rate. However, it is not clear whether adaptations in forgetting rate were due to an explicit awareness of changes in probing hazard, or whether adaptations in forgetting rate were driven by implicit knowledge, like in statistical learning (Sherman et al., 2020). Fortunately, Experiment 3 was ideally suited to answer this question. At the end of the experiment, participants were first asked whether they noticed anything had changed halfway through the experiment, and if so, to write a short description of what had changed. In total, 57% of participants did not notice anything had changed, and those who indicated that they did notice something were all unable to identify what had changed. Then, participants were asked whether they had noticed that they needed to remember the color for longer/shorter in one half of the experiment, and shorter/longer in the other half. Most participants (62%) indicated that they did not notice the change in overall retention interval. Finally, participants were prompted to guess the order of probing hazard conditions with a forced two-alternative question. Overall, participants could not correctly indicate the order of conditions, regardless of whether they said they had noticed the change (50% correct) or not (57% correct). Notably, participants were biased towards indicating that retention intervals became longer in the second half of the experiment (72%), possibly due to time-on-task effects. In sum, participants did not spontaneously report the switch in probing hazard, (mostly) did not say that they had noticed the change, and could not accurately report the order of conditions when prompted. In sum, it seems that the adaptations in forgetting rate that we observed were not mediated by explicit awareness of how quickly information became outdated.

## Discussion

Humans can use explicit cues to forget information (Dames & Oberauer, 2022; Williams et al., 2013; Williams & Woodman, 2012), freeing up working memory capacity, but these cues might be rare in real-life situations. We hypothesized that humans implicitly tune their rate of forgetting to the temporal contingencies of whether they need an item. Indeed, we found that information was forgotten more quickly when information became irrelevant more quickly.

Our findings are in stark contrast to accounts that regard forgetting in working memory as an unfortunate limitation of cognitive or neural processes (Jonides et al., 2008). Instead of treating working memory forgetting as “decay” (Ricker et al., 2016), “sudden death” (Zhang & Luck, 2009) or “interference” (Souza & Oberauer, 2015), our findings suggest that forgetting in working memory is more adaptive than those labels signify. To be clear, some forms of forgetting are likely the result of systematic limitations of neural or cognitive processes. However, those incidental forms of forgetting might have little to do with the adaptive forgetting mechanisms we demonstrated here, and exist alongside the mechanisms demonstrated with the current set of experiments. For instance, forgetting in typical working memory experiments may have been systematically underestimated because of an increasing probing hazard (also see, e.g., Muter, 1980). Instead, we show substantial forgetting for a single item in working memory with decreasing probing hazard, comparable to forgetting at higher set sizes (e.g., Pertzov et al., 2017; Souza & Oberauer, 2015; Zhang & Luck, 2009). This also speaks against accounts that claim forgetting results exclusively from competing items held in working memory (Pertzov et al., 2017), or from interference from concurrent tasks (Barrouillet et al., 2011). In our experiments, only a single item was held in working memory, and there was no concurrent task.

One source of interference that was still possible in our task was proactive interference. Items previously held in working memory may interfere with currently maintained items (Keppel & Underwood, 1962). In “decreasing” blocks, items were probed in more rapid succession, which might lead to more proactive interference (Souza & Oberauer, 2015). However, some patterns in our data suggest that proactive interference cannot fully explain our findings. For instance, previous studies have found that proactive interference impacts visual working memory performance at all retention intervals (Shoval & Makovski, 2021; Souza & Oberauer, 2015). In contrast, we did not observe such a cost for the shortest retention interval. If anything, participants performed better in “decreasing” blocks at the briefest retention interval than in “increasing” or “flat” blocks. While the effect of proactive interference was likely minimal in our experiments, future studies should attempt to experimentally (e.g., by having non-overlapping item presentations; Makovski, 2016) or statistically control for proactive interference (Bays et al., 2009; Taylor et al., 2023).

We believe that the main “rationale” of adaptive forgetting is to optimize the use of limited working memory capacity. In light of this rationale, adaptive forgetting should be systematically related to set size and capacity. For instance, if a participant’s capacity is three items, we expect quite drastic adaptive forgetting if they are shown two items at a time. The participant would need to drop at least one of the current items in order to optimally prepare encoding for the next two items. Therefore, we might expect individuals with low capacity to have a larger need to forget. Alternatively, individual differences in working memory capacity may stem from suboptimal forgetting mechanisms. Individuals with low capacity may be less able to suppress obsolete items, overloading their working memory in the process (cf. Engle, 2002). Future research should aim at dissociating these competing hypotheses.

In terms of underlying mechanisms, two opposing accounts might explain how information is adaptively forgotten. First, by the time an item is likely obsolete, participants might terminate active maintenance of that memory item, exposing it to decay (Barrouillet et al., 2011) or interference (Souza & Oberauer, 2015). According to this hypothesis, it is the disengagement of active maintenance processes that implements adaptive forgetting. For instance, Dames and Oberauer (2022) concluded that directed forgetting in working memory is implemented by boosting the representations of to-be-remembered items, while leaving to-be-forgotten items untouched. Another possibility is that by the time an item is likely obsolete, participants actively inhibit the to-be-forgotten item. Accordingly, participants employ an active mechanism to remove to-be-forgotten items from working memory (Lewis-Peacock et al., 2018). Several findings suggest that active inhibitory mechanisms operate to remove information from working memory after explicit forgetting cues (Festini, 2020), to resolve proactive interference in working memory (Jonides et al., 1998), and to settle competition between items during working memory retrieval (Kang & Choi, 2015). Furthermore, it has been suggested that active forgetting from working memory is accomplished by “unbinding” an item from its context (e.g., spatial position, temporal order; Lewis-Peacock et al., 2018). More work is needed to uncover which of these mechanisms underlies adaptive forgetting rate in working memory.

We observed that the adaptations in forgetting rate in our experiments can be subtle. Experiment 2 did not show a significant effect of probing hazard, and Experiment 3 did not find a difference in the first half of the experiment. There are several factors that may have limited our power to detect adaptive forgetting rates. For instance, the number of trials that could be used to compute forgetting rates was limited as a result of the manipulation of hazard drop. Future studies should attempt more powerful replications. We recommend including more participants for between-participant comparisons, using more sensitive measures of working memory performance (e.g., Honig et al., 2020), and boosting the effect, for instance, by increasing the hazard drop or possibly the set size.

In conclusion, we found evidence that humans can implicitly tune their rate of forgetting in working memory to the rate at which information becomes obsolete. Specifically, when the probability that a piece of information would be probed decreased over time, participants forgot that information more quickly. This may serve the purpose of freeing up capacity in environments where the relevance of information is ever-changing. Notably, participants were unaware of changes in the rate at which information became obsolete, suggesting that adaptations in forgetting are largely implicit. Our findings also raise some questions for future research, such as the relationship between an individual’s working memory capacity on adaptive forgetting, the fate of adaptively forgotten memories, and the active or passive mechanisms by which information is adaptively cleared from working memory.

## Data Availability

Data and materials for all experiments are available on the Open Science Framework website (https://osf.io/chxp3/)

## Code availability

Experiment and analysis code are available on the Open Science Framework website (https://osf.io/chxp3/)
